# Hydrodynamics of the fast-start caridoid escape response in Antarctic krill, *Euphausia superba*

**DOI:** 10.1038/s41598-023-31676-8

**Published:** 2023-04-02

**Authors:** A. A. Connor, D. R. Webster

**Affiliations:** 1 School of Civil and Environmental Engineering, Georgia Institute of Technology, Atlanta, GA 30332-0355 USA; 2 George W. Woodruff School of Mechanical Engineering, Georgia Institute of Technology, Atlanta, GA 30332-0405 USA

**Keywords:** Biological physics, Fluid dynamics

## Abstract

Krill are shrimp-like crustaceans with a high degree of mobility and variety of documented swimming behaviors. The caridoid escape response, a fast-start mechanism unique to crustaceans, occurs when the animal performs a series of rapid abdominal flexions and tail flipping that results in powerful backward strokes. The current results quantify the animal kinematics and three-dimensional flow field around a free-swimming *Euphausia superba* as it performs the caridoid escape maneuver. The specimen performs a single abdominal flexion-tail flip combination that leads to an acceleration over a 42 ms interval allowing it to reach a maximum speed of 57.0 cm/s (17.3 body lengths/s). The krill’s tail flipping during the abdominal closure is a significant contributor to the thrust generation during the maneuver. The krill sheds a complex chain of vortex rings in its wake due to the viscous flow effects while the organism accelerates. The vortex ring structure reveals a strong suction flow in the wake, which suggests that the pressure distribution and form drag play a role in the force balance for this maneuver. Antarctic krill typically swim in a low to intermediate Reynolds number (*Re*) regime where viscous forces are significant, but as shown by this analysis, its high maneuverability allows it to quickly change its body angle and swimming speed.

## Introduction

The caridoid escape response, often referred to as the tail flipping mechanism, is a fast-start escape response that is unique to malacostracan crustaceans. This suborder of crustaceans has an elongated abdomen that allows for animals to combine a rapid abdominal flexion with a tail flipping motion that leads to an acute increase in the animal’s velocity. Most of the studies of this maneuver have been on large benthic and epibenthic crustaceans such as lobsters^1–3^, crayfish^4–6^, and shrimp^7–9^. Correspondingly, there is limited previous work quantifying the kinematics and hydrodynamics of the caridoid escape response in krill (see Supplemental video 1).

Antarctic krill, *Euphausia superba*, are estimated to represent the largest biomass for any species on the planet^10^. In the Southern Ocean, they can be up to 50 percent of total zooplankton biomass in the surface ocean layer^11, 12^ and 33 to 90 percent of the diet of larger marine carnivores^13^. Because of their importance in the Southern Ocean, Antarctic krill are one of the most studied pelagic animals with a wide range of documented studies on krill fisheries^14^, distribution across the Southern Ocean^15–17^, predator diet^18–20^, changes due to ocean acidification and global warming^21–23^, and swarming and schooling behavior^24–26^.

Quantifying the locomotion and flow characteristics of essential zooplankton, such as Antarctic krill, leads to better understanding of patterns and changes within the global marine food web. *Euphausia superba* often have a lifespan of 4–7 years in their natural environment despite constant predation, harsh environmental conditions, and food scarcity in the Southern Ocean^27, 28^. One proposed reason for *E. superba*’s longevity is that as these small crustaceans reached adulthood, they transition into animals of high locomotion, agility, and maneuverability despite the viscous flow effects of their flow regime^29^. Krill use their five sets of pleopods, as well as their tail, to achieve a high level of maneuverability^30^. As a result, there is a growing field with the objective of quantifying the pleopod kinematics and locomotion^30, 31^, schooling behavior ^24, 26^, and propulsion hydrodynamics^32, 33^ of Antarctic krill.

Relatively less is known about the caridoid escape or tail flipping maneuver. The tail flipping maneuver is reported as a common tactic used by Antarctic krill to escape close contact with predators^34^. There have been several reported observations of this mechanism in the field^34, 35^ and laboratory^29^. Most studies, to date, of the caridoid escape maneuver in *E. superba* have been qualitative, and none have looked at the flow characteristics. However, Kils measured the drag coefficient in the tail flipping orientation and reported that this orientation is the most streamlined in order to reduce water resistance and maximize lift to efficiently escape predators^36^.

The tail flipping behavior has been studied in other crustaceans. For instance, Arnott et al*.* found that the kinematics of the tail flipping mechanism varied with animal size in the brown shrimp, *Crangon crangon*^7^. As the animal’s length increased, the duration of the tail flip increased. Daniel and Meyhöfer hypothesized, using conservation of momentum principles, that a hydrodynamic “squeeze” force is produced by the expulsion of water by the abdomen and cephalothorax during the abdominal flexion part of the maneuver in carridean shrimp^37^. Hunyadi et al*.* compared the escape response efficiency in male versus female crayfish (*Faxonius rusticus*)^6^. When quantifying the fluid motion around the tail during the abdominal closure, they found no evidence of a hydrodynamic “squeeze” force in the flow field. Instead, they observed the formation of a tip vortex along the tail during the abdominal closure, which generated significant thrust to rapidly accelerate the animal. Hunyadi et al*.* additionally observed that the animals also moved their pleopods during the tail flip maneuver, which also contributed to the thrust generated by the crayfish^6^. Whereas previous studies of the caridoid escape maneuver investigated how thrust is generated during the tail flipping and abdominal closure, there have been no studies examining the flow characteristics after these crustaceans perform this maneuver. In part, this is due to the challenge of capturing the unpredictable maneuver in untethered animals.

This study presents the first quantitative report on the flow characteristics and locomotion of the tail flipping escape mechanism in Antarctic krill, *E. superba*. The surrounding fluid motion is quantified using the three-dimensional particle image velocimetry (PIV) technique and the kinematics data are compared to that of malacostracan crustacean species of similar size. The results provide novel insight on the hydrodynamics of the wake of this escape mechanism, in which *E. superba* produces a series of complex vortex rings that are believed to help reduce form drag and preserve the animal’s increased velocity following the abdominal closure.

## Methods

Antarctic krill (*E. superba*) were collected from Palmer Deep (64°57′ S, 64°24′ W) and Boyd Strait (62°50′ S, 62°00′ W) in the Southern Ocean on-board RV Laurence M. Gould using a plankton net with 2 × 2 m rim size and 500 μm mesh size. After collection, animals were held in large buckets of seawater with salinity of 34.6 parts per thousand (ppt), stored at 0 °C, and transported to Palmer Station (Anvers Island, Antarctica; 64°46′S, 64°03′W). Seawater was obtained from the coastal ocean waters near Palmer Station, filtered, and placed in a glass test tank (10 × 10 × 12 cm, W × D × H). The test tank was filled to a height of 10 cm.

Because Antarctic krill typically reside in waters with a temperature of ~ 0 °C, the test tank was submerged in a transparent acrylic glycol–water bath tank (30 × 30 × 30 cm). This bath tank contained a closed-loop heat exchanger coil that maintained the temperature of the test tank at 0 °C throughout the experiment. The entire apparatus was located in a temperature-controlled room at 10 °C. To track the motion of the fluid around the animal, the seawater in the test tank was seeded with 20 µm polyamide (1.03 g/cm^3^) tracer particles (Arkema Group). These particles are inert to krill and illuminate well under near infrared light.

For data capture, the specimen was filmed at 500 frames per second using a portable, infrared tomographic Particle Image Velocimetry (tomo-PIV) system that was specially designed to study zooplankton flows^38^. For reference, Adhikari et al.^38^ present detailed experimental apparatus schematics of the system, which is described briefly here. In this set-up, the recording was captured using four synchronized high-speed Phantom v210 cameras (Vision Research Inc., 1280 × 800 pixels) with 105 mm lenses (Nikon). The cameras were positioned approximately at a 30° half angle from the targeted viewing area with respect to the horizontal and vertical axes. To limit off-axis distortion, the depth of field and positioning of camera lenses were adjusted by setting the aperture of the lenses to between f11 and f16 and adjusting the Scheimpflug adapters (LaVision GmbH) to align the lenses to the same viewing plane.

Antarctic krill are photo-sensitive to optical wavelengths^39^. Therefore, near infrared lasers (wavelength 808 nm, CrystaLaser Inc.) were used to illuminate the interrogation region because it has been shown that zooplankton do not alter their swimming behavior when exposed to infrared light^33, 40, 41^. The lasers were positioned on opposite sides of the test tank in the same plane to reduce shadowing during imaging due to the presence of the animal within the illuminated volume. Each laser was collimated, expanded, and tapered to an interrogation volume of roughly 36 cm^3^ (6 × 4 × 1.5 cm; X × Y × Z).

### Kinematics processing

The caridoid escape maneuver recorded in this study was induced by the specimen approaching and sensing a stationary glass rod in the experimental tank. The specimen’s kinematics were quantified by digitally tracking 14 points along the body (Supplementary Fig. S1) in three of the four synchronized images for each time point (performed semi-manually). A direct linear transform (DLT) software was applied to triangulate the three-dimensional location of each point using the same calibration plate images employed for the tomo-PIV measurements^42^. Similar approaches for quantifying kinematics have been used for several species of krill, and the procedure results in minimal noise in the velocity estimate due to the high recording resolution, high recording frame rate, excellent animal visibility, and robust internal DLT calibration process^30, 31^. In this study, the points chosen were one point at the eye (point 1), one point along the each of the five segments of the dorsal region (points 2–6), one point at the base of each pleopod pair (points 10–14), one point at the base of the tail (point 7), and two points at the end of the tail (points 8–9) (Supplementary Fig. S1).

### Flow field processing

A mapping function for the interrogation volume was created in DaVis 8.4 (LaVision GmbH) by positioning a calibration plate at six unique positions in the direction that was orthogonal to the viewing plane of the cameras. This mapping function was corrected by using the self-calibration procedure in DaVis 8.4^43^. This method significantly reduces disparity errors, which allows the 3D algorithms to locate particles more accurately. In this study, the particle intensity volumes were reconstructed for each time frame using the MLOS-CSMART algorithm.

A visual hull technique was applied to remove the reflected light imaged in the volume region associated with the animal position, which prevents artifacts in the flow field and vector contamination near the organism^38, 41, 44^. Then, a volume of three-component velocity vectors was calculated by cross-correlating the particle intensity volume pairs at each 0.002 s interval. The velocity fields were combined with the animal’s visual hull for visualization and further analysis.

## Results

**Figure 1:**
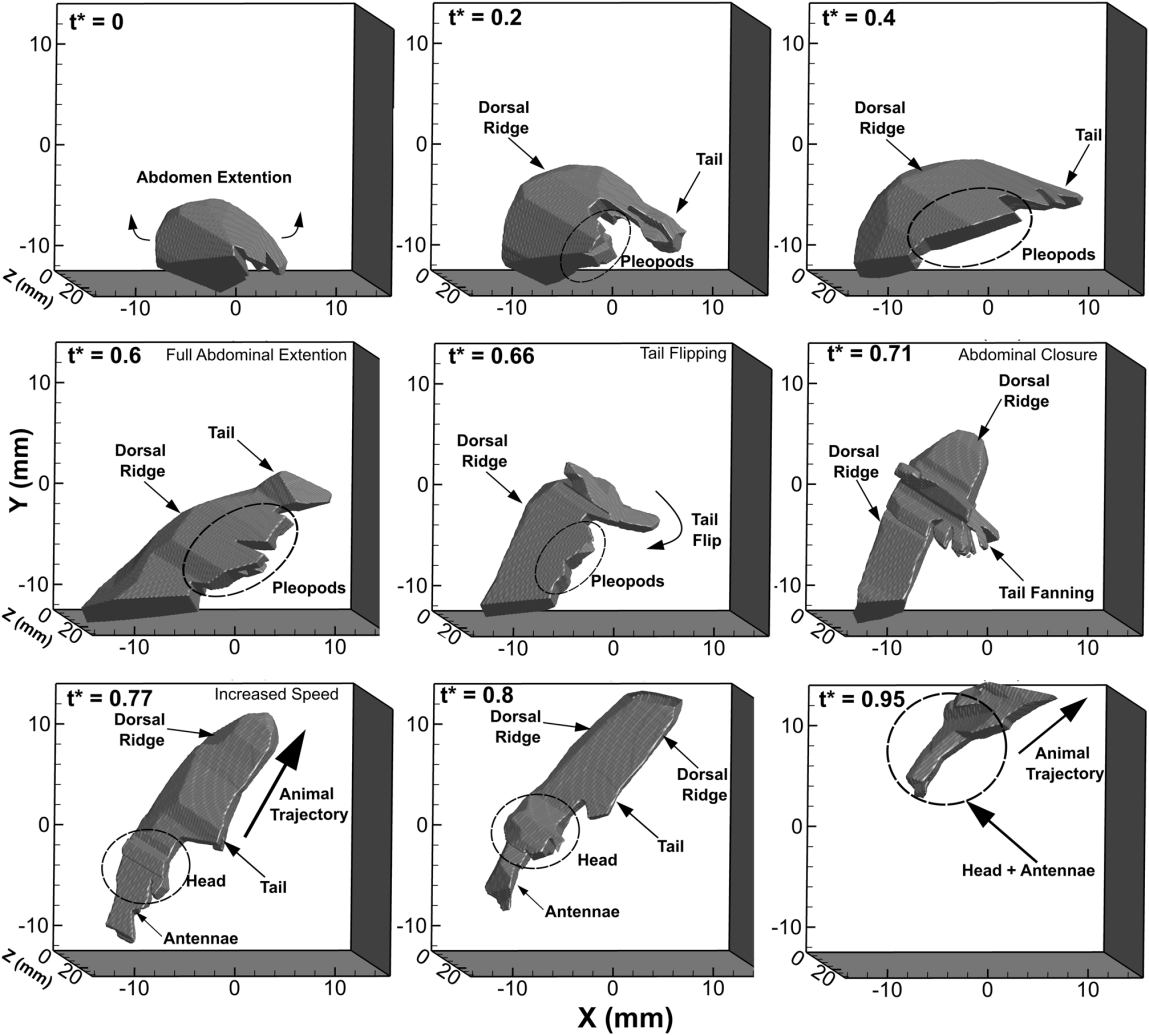
Time sequence of the reconstructed 3D visual hull of *E. superba* performing the caridoid escape response where t* is time non-dimensionalized by the total stroke period, which is 322 ms. The animal starts in a closed body position, fully extends the ventral cavity and then performs a rapid abdominal flexion and tail flip that leads to rapid acceleration of the animal to the upper right section of the viewing window. Note that the head and antennae of the animal are partially out of frame of the viewing window from t* = 0 to t* = 0.71 as well as the tail and dorsal ridge at t* = 0.95 leading to an exclusion of these body parts during the reconstruction of the 3D visual hull.

Figure 1 shows a time series of the 3D visual hull of *E. superba* performing the caridoid escape maneuver, which orients the reader to the 3D spatial nature of the flow field analysis, and Supplementary Fig. S2 shows an image sequence from one of the four camera perspectives at the same nine time points. The total stroke period is 322 ms, which is used to non-dimensionalize the time variable. In the figures, it can be seen that the animal begins the maneuver in a closed body C-shaped position on the left side of the view window (in front of the illuminated fluid volume in the tank). The animal rapidly extends its abdomen to create more space between its head and tail. At t* = 0.6, the animal begins to perform a sharp abdominal flexion and tail flipping motion that brings the tail and head together forming a body crescent shape. This motion occurs between t* = 0.6 and t* = 0.8, which corresponds to a time interval of 42 ms. In the frames that showcase the tail flip (t* = 0.60 and t* = 0.71), there is evidence of the animal fanning its tail during the abdominal closure, which is especially prominent in the visual hull panels in Fig. 1. During the abdominal closure, the animal rapidly accelerates backwards leading with the central dorsal ridge of its body and with its head and antennae trailing. The animal then rapidly propels upwards until it travels out of the observation region.

**Figure 2:**
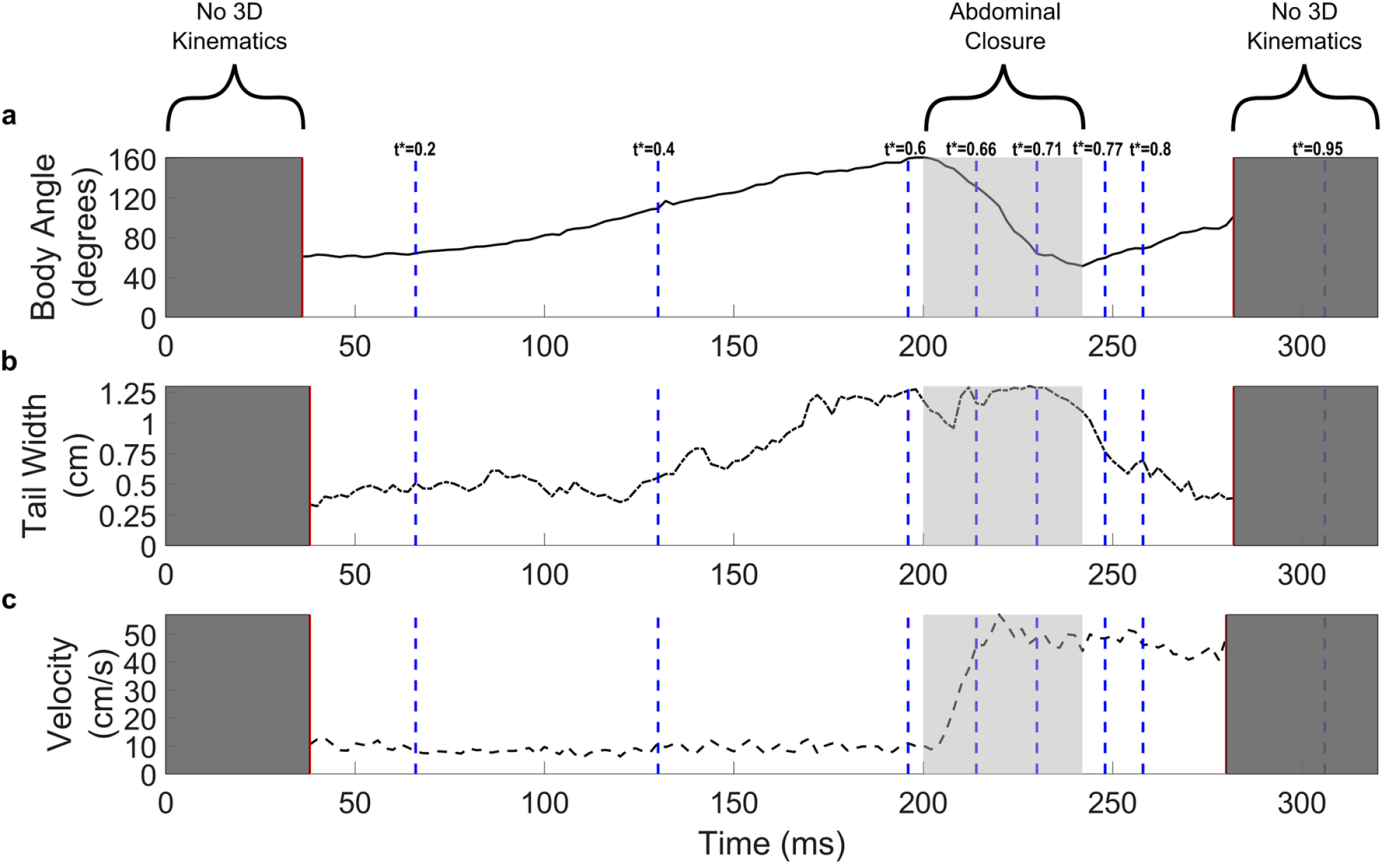
Time records of (**a**) body angle, (**b**) tail width, and (**c**) velocity of *E. superba* as it performs the caridoid escape maneuver. Body angle was measured by calculating the angle between 2 vectors created using three points along the body: the eye, the middle of the dorsal side of the abdomen, and the base of the tail (i.e., points 1, 4, and 7, respectively, in Supplementary Fig. S1). The tail width was measured by calculating the length between the left and right tips of the tail. The velocity was calculated by ensemble averaging the motion of all 14 points defined in Supplementary Fig. S1. The dark grey shaded regions represent portions of the recordings where 3D kinematics were not able to be calculated. The light grey shaded region corresponds to the abdominal closure portion of the caridoid escape maneuver. The dashed blue lines indicate the time points of the images displayed in Fig. 1 and Supplementary Fig. S2.

Figure 2 shows the time series of body angle, tail width, and velocity as *E. superba* performs the caridoid escape maneuver. Due to the camera perspectives that are needed for the 3D position reconstruction, a full kinematic data set was captured only between 36 and 284 ms (t* = 0.11 to t* = 0.88). Each panel contains a set of blue dashed vertical lines that correspond to the frames showcased in Fig. 1 and Supplementary Fig. S2. The body angle and tail width both gradually increase over time until about 200 ms (t* = 0.62) and this time interval corresponds to the extension of the abdominal region. Starting at 200 ms, the body angle shows a rapid drop, which is associated with the abdominal closure/tail flip interval (Fig. 2a). This abdominal closure interval is marked in Fig. 2 as the region shaded in light grey.

The animal velocity increases rapidly after this abdominal closure interval begins (Fig. 2c). Although the body angle decreases during this closure interval (Fig. 2a), the tail width remains at its maximum width until the abdominal closure interval is complete (Fig. 2b). This maximizes the surface area of the tail during the maneuver. The tail width begins to decrease after the abdominal closure interval (Fig. 2b), and the velocity plateaus at the higher speed (Fig. 2c). The maximum velocity produced by this maneuver is 57.0 cm/s, which corresponds to 17.3 body lengths/s (BL/s), and this peak in velocity occurs roughly halfway through the abdominal closure period. Using the body length as the characteristic length scale, the Reynolds number (*Re*) for this maneuver is in the range of 1800–11,000, which indicates that *E. superba* is maneuvering in the intermediate *Re* flow regime. The maximum acceleration for the maneuver was calculated (by central difference) to be 273 m/s^2^, which is roughly 28 times the acceleration of gravity.

**Figure 3:**
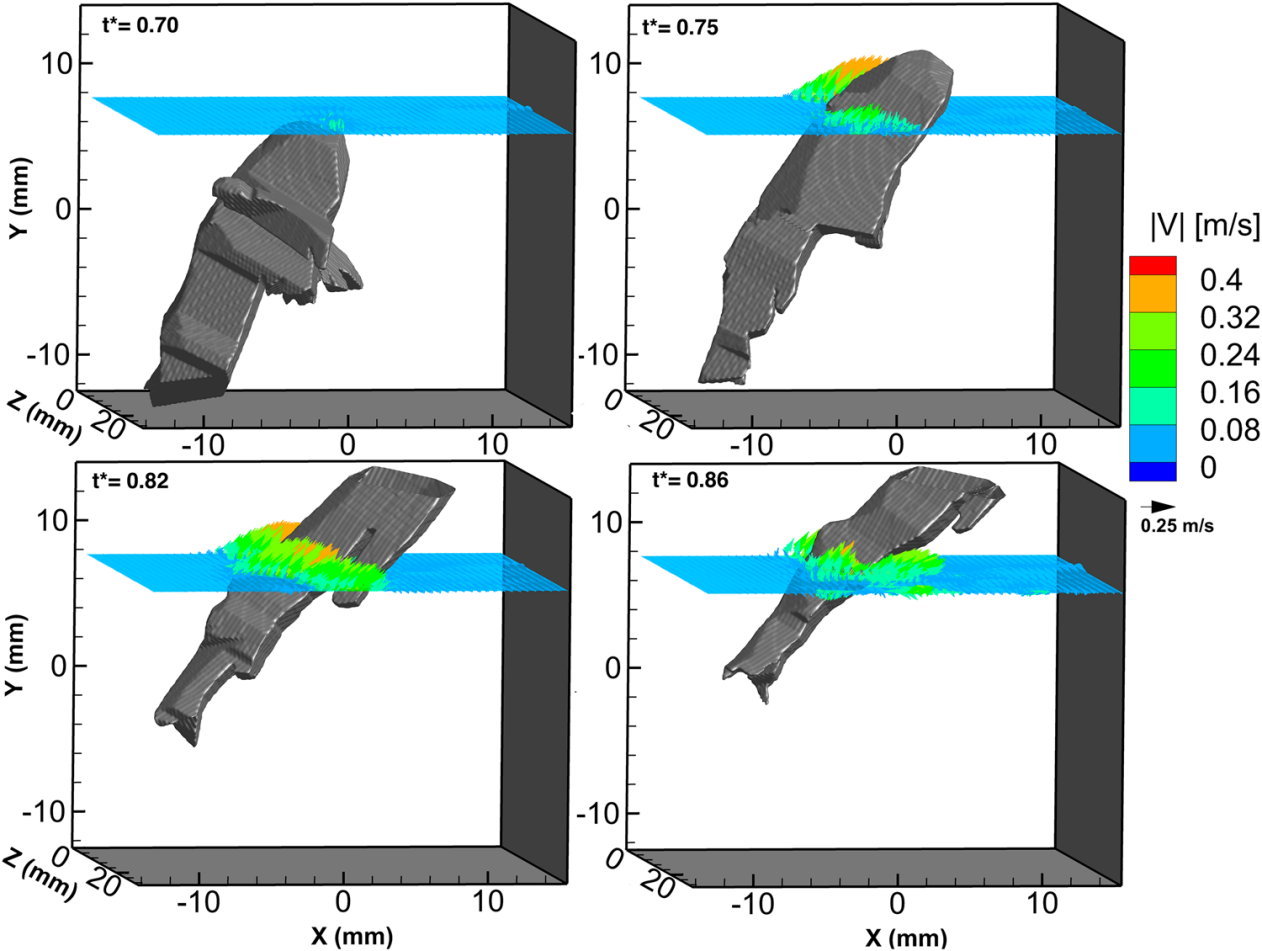
Time sequence of the fluid velocity field in a XZ plane while *E. superba* performs the tail flip and abdominal closure and accelerates to the upper right region of the viewing window. The non-dimensional time point is labeled in each frame. The velocity vectors are colored to indicate the fluid velocity magnitude, as defined in the legend. At time point t* = 0.70, the animal is executing the tail flip, and during the time period t* = 0.75–0.86, the krill has completed the abdominal closure and its velocity is elevated.

Detailed 3D flow analysis was performed on the animal and wake after the tail flip and rapid acceleration of the animal, which is the time interval that had the best illumination and camera perspectives to produce accurate 3D flow field reconstruction. Note that the orientation of the animal in relation to the cameras was not ideal for detailed analysis during the abdominal closure period. Figure 3 shows a time sequence of a slice of the flow field in a XZ plane as the krill moves at its maximum velocity. The grey object is the visual hull of the krill for spatial reference. The orientation of the volume shows a ventral view of the animal as it completes the tail flip and accelerates through the XZ plane. The vectors are oriented to indicate the direction of the fluid flow at that spatial point along the plane. From the time sequence of the krill moving through the fluid plane, the fluid near the surface of the animal is being dragged upwards as the animal moves. The thickness of this layer of elevated fluid velocity around the animal is roughly 3 mm.

**Figure 4:**
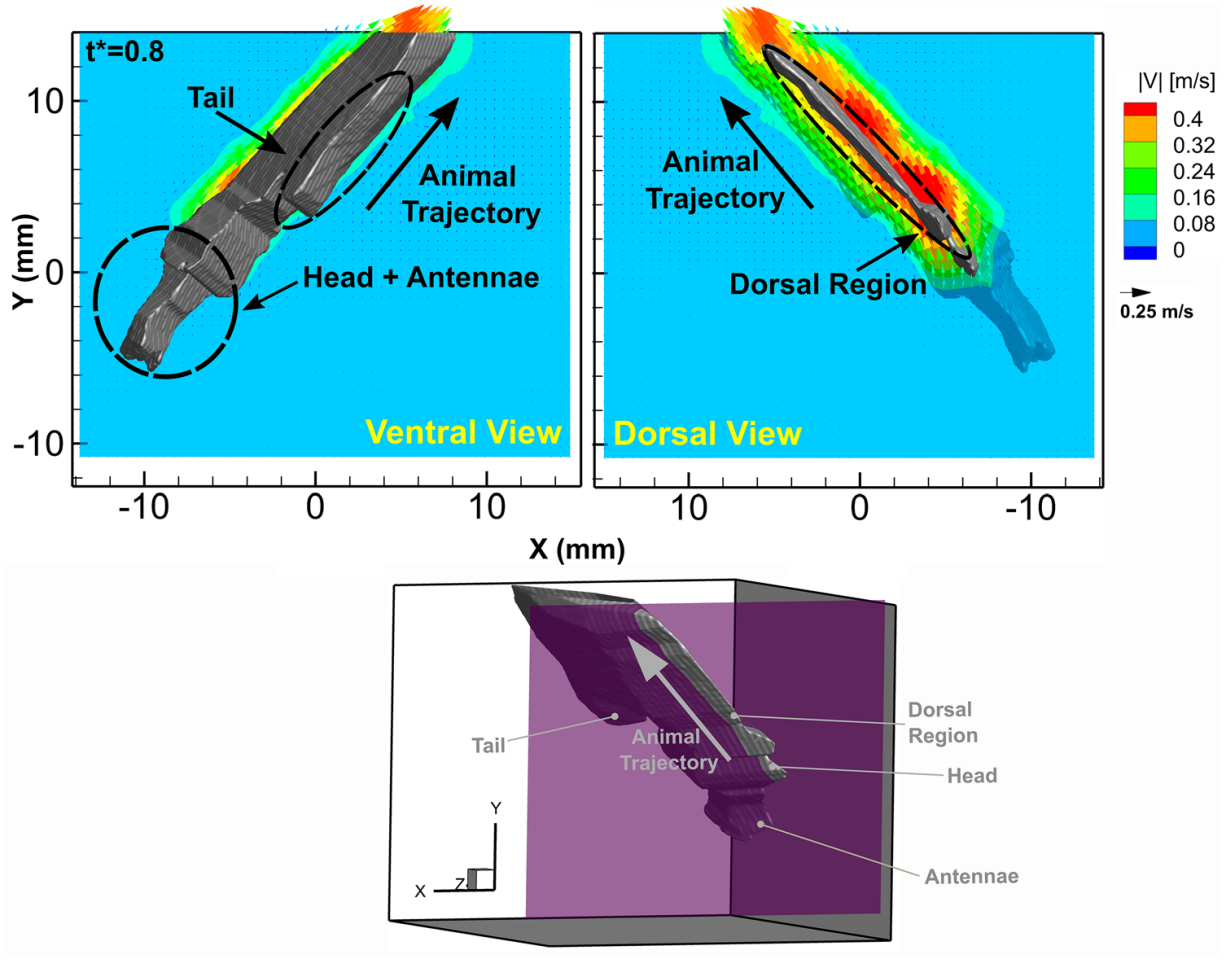
Snapshot at t* = 0.8 of the fluid velocity field in a XY plane (for Z = 5.86 mm) bisecting the dorsal ridge of the animal while *E. superba* moves through the plane after the abdominal closure and increase in velocity. The plane is shown from both the ventral view and the dorsal view, where the dorsal ridge of the animal is visible. The velocity vectors are colored to indicate the fluid velocity magnitude, as defined in the legend. The XY plane was extracted from the full 3D vector flow field. To help to orient the planar field, a small 3D reconstruction of the slice orientation with respect to the 3D visual hull is shown in the bottom center of the figure.

Figure 4 shows a XY plane of the flow field tangent to the dorsal ridge of the krill revealing the flow field when the animal is moving at high velocity (at t* = 0.8). As seen in the slice orientation inset, this plane bisects the dorsal ridge of the animal. The flow field is shown from two spatial orientations: a ventral view on the left where the undercarriage of the animal is facing outwards from the page and a dorsal view on the right where the back or dorsal ridge of the animal is facing out from the page. The grey structure is the visual hull of the animal where the dashed circles highlight the location of anatomical features including the head and tail in the ventral view and the dorsal ridge in the dorsal view. The direction of the animal trajectory is marked by the black arrow.

With reference to Fig. 1 and Supplementary Fig. S2, at the time point shown in Fig. 4, the animal has already performed the tail flip and fully closed its body and tail. At this time point, the animal is traveling at a velocity of 50 cm/s (15 BL/s; Fig. 2c). The surrounding fluid reaches maximum speed of greater than 40 cm/s near the surface of the animal moving in the direction of the animal’s motion (Fig. 4). Although the fluid at the animal surface is 50 cm/s due to the no-slip condition, 40 cm/s is the largest velocity that can be resolved using this technique due to resolution of the flow field and a small amount of vector contamination near the surface of the animal due to the animal masking step. The high velocity region is localized to near the surface of the animal. The fluid velocity decreases quickly in the direction orthogonal to the visual hull surface and is close to zero outside a fluid layer of 3 mm, which is consistent with the layer thickness shown in Fig. 3.

**Figure 5:**
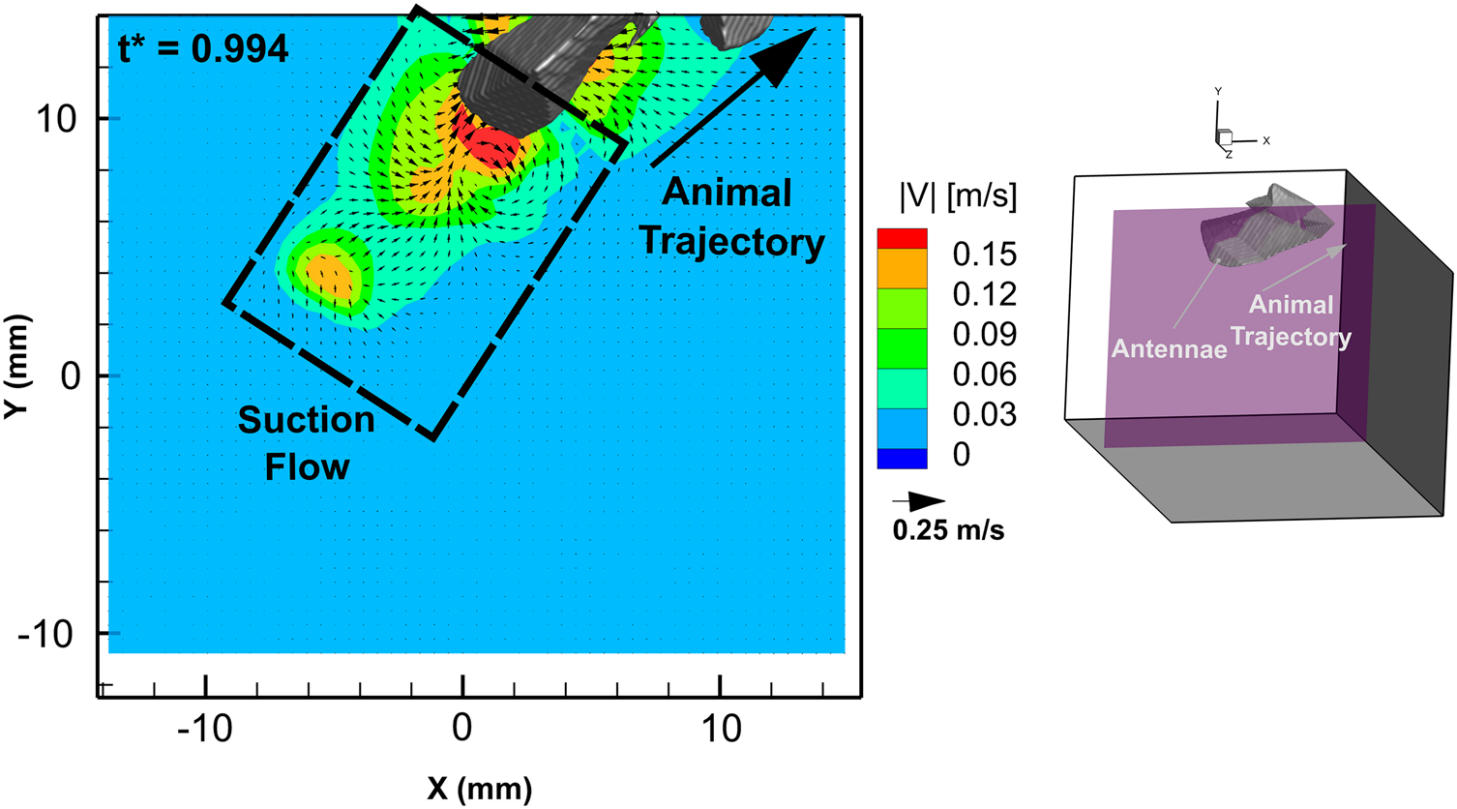
Snapshot at t* = 0.994 of the velocity field in a XY plane (for Z = 4.86 mm) bisecting the dorsal region of the animal while *E. superba* moves through the plane after the abdominal closure and increase in velocity. The XY plane was extracted from the full 3D vector flow field. The color contours correspond to the fluid velocity magnitude. To help to orient the planar field, a small 3D reconstruction of the slice orientation with respect to the 3D visual hull is shown on the right.

Due to the mechanics of the caridoid escape maneuver motion, the wake of the animal happens to be adjacent to the head and antennae, instead of the tail. Figure 5 shows a planar snapshot of the wake around the end of the antennae. This plane shows contours of the fluid velocity magnitude, and as seen from the inset figure containing the slice orientation and the visual hull; this 2D slice is positioned to bisect the center of the antennae. In the region of the flow field that surrounds the antennae, a small volume of fluid behind the animal flows with the animal as it moves towards the upward right of the viewing window. At the edges of the high velocity regions in the wake, the flow appears to have rolled up into vortical structures.

**Figure 6:**
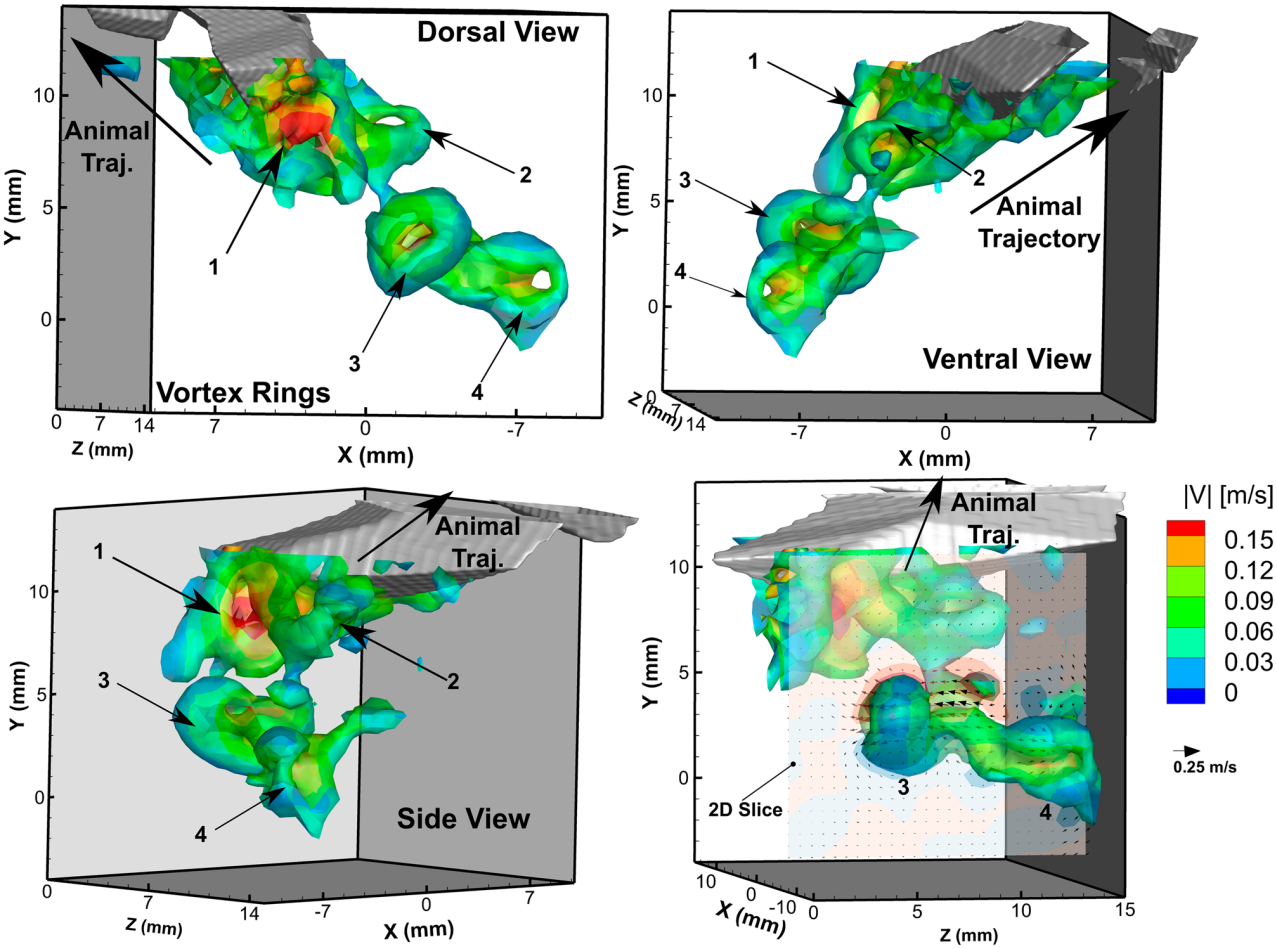
Volumetric isosurface of vorticity magnitude surrounding the antennae and its wake at t* = 0.994 from three perspectives, and a 2D slice of the YZ plane with corresponding velocity vectors bisecting vortex ring 3. The isosurface corresponds to vorticity magnitude of 65 s^-1^, and the color contours represent the fluid velocity magnitude along the isosurface.

Figure 6 shows the volumetric isosurface of the vorticity magnitude with overlayed contour color map of the fluid velocity magnitude at the isosurface location from several spatial perspectives. The isosurface reveals a complex vortical structure in the wake of the animal’s antennae after the abdominal closure and increase in its velocity. From this figure, which was extracted at t* = 0.994, four distinct vortex rings are observed in the wake, which are labeled in each of the 3 spatial views. From the side view and dorsal view, the vortex rings appear connected and staggered with distance from the animal. The center of each vortex core has a high velocity region, whereas the outer edges of the rings are relatively smaller in velocity. This indicates that there is a jet of fluid passing through the center of each vortex ring. The bottom right panel in Fig. 6 shows a 2D slice that was overlayed to bisect vortex ring 3 showing the jet of fluid through the vortex ring center.

**Figure 7:**
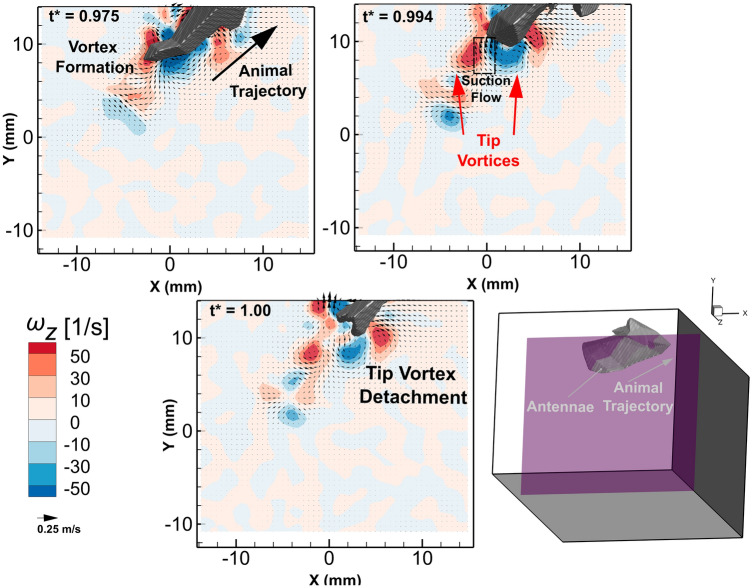
Time series of the *z*-component of vorticity, ${\omega }_{z}$, field in a XY plane (for Z = 6.36 mm) centered along the antennae and its wake while *E. superba* moves through the plane at maximum speed. The XY plane was extracted from the full 3D vector flow field. A small 3D reconstruction of the slice orientation with respect to the 3D visual hull is shown in the bottom right.

A time sequence of these vortical structures provides insight on the shedding mechanics from the antennae region of the animal. Figure 7 shows a time series of a 2D slice of the z-component of vorticity, $\omega_{z}$, and a ventral view of the animal’s antennae. As shown in the Fig. 7 inset, the slice bisects the center of the antennae of the animal. In this time series, the regions of positive and negative $\omega_{z}$ build on either side of the antennae due to fluid shearing adjacent to the surface of the animal. A tip vortex pair forms in the wake of the antennae. At time point t* = 0.994, the tip vortices are fully formed and there is a small jet flow with fluid moving in the direction of the animal motion. This flow pattern is consistent with the presence of vortex ring 1 (shown in Fig. 6) at the same time point. Detached vortex cores are shown in the wake of the antennae at t* = 1 in Fig. 7.

**Figure 8:**
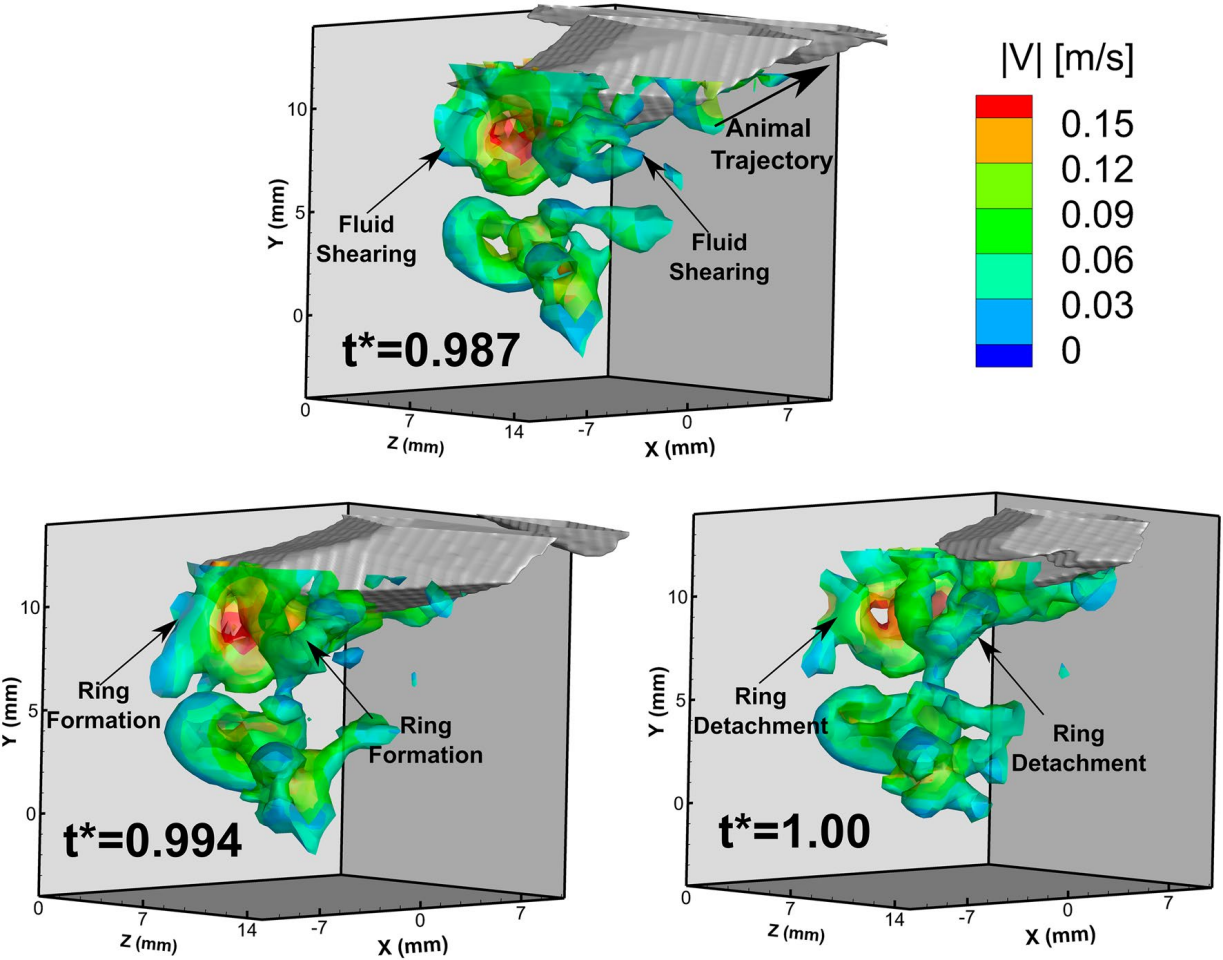
Time series of the volumetric isosurface of vorticity magnitude surrounding the tip and wake of the animal’s antennae. At these time points, *E. superba* has already performed its abdominal closure and increased its velocity. This time series shows the formation and detachment of vortex rings 1 and 2 from the surface of the animal’s antennae. The isosurface corresponds to vorticity magnitude of 65 s^-1^, and the color contours represent the fluid velocity magnitude along the isosurface.

Figure 8 is a time series of the 3D vortical structure formation in the wake of the antennae. Each time point shows the volumetric isosurface of vorticity magnitude with a color contour map of fluid velocity magnitude along the isosurface. The 3D plot is spatially oriented to show the side view of the animal in each panel (corresponds to the side view panel in Fig. 7). This time series shows the formation of the toroidal vortices 1 and 2 that separate from the end of the antennae. At t* = 0.987, the wake vortices are forming at the tip of the antennae due to fluid shearing. These vortex rings separate from the tip of the antennae to form the series of vortical structures in the wake.

## Discussion

### Flow features

The hydrodynamics of the caridoid escape maneuver of an untethered free-swimming crustacean have not been previously investigated. The current work provides high spatial and temporal resolution of the three-dimensional flow field induced by a caridoid escape response in the Antarctic krill, *E. superba*. An interesting observation from the results is that although the relative speed of the animal is large for this maneuver when normalized to the body length (17.3 BL/s), the extent of the wake disturbance behind the antennae is relatively small, measuring just under 1 cm (i.e., 0.3 BL). In contrast, the wake of Antarctic krill swimming with metachronal padding at transitional speeds between hovering and fast forward swimming (i.e., less than 2 BL/s) extends roughly 0.5 BL vertically and roughly 3 BL horizontally behind the animal^32^. However, for the current data, the wake disturbance induced by the animal extends only around 0.3 BL from the tip of the antennae after the animal performs the tail flip and increases its speed. This suggests that there is minimal momentum transfer or loss from the animal to its environment in the glide phase, which is also supported by the constant animal speed after the abdominal closure interval (Fig. 2c). This result could be because the animal has been reported to be relatively streamlined in this C-shaped geometry while performing the maneuver, with a reported drag coefficient of around 0.3^36^.

The results also reveal that there is a boundary layer of fluid being dragged with the animal as it propels itself backwards, as shown in Figs. 3 and 4. The layer thickness is larger than expected based on boundary layer theory and, rather, may be explained by the Darwin drift mechanism, in which a hydrodynamic mass is dragged along with the moving object^45^. This form of fluid boundary layer has not been reported before in the 2D hydrodynamics of metachronal swimming in Pacific krill^32^ or in the 3D hydrodynamic study of hovering in Antarctic krill^33^. This phenomenon of a boundary layer around an animal as it performs the escape response also has not been reported for any other hydrodynamic study of this maneuver in other crustaceans. One reason for this may be that many hydrodynamics studies on live aquatic animals are performed on tethered animals^6, 46^ or do not include flow measurements if the animals are free swimming^1, 7, 36, 47^. A prominent boundary layer has only been reported for smaller mesozooplankton, such as copepods, which perform robust high velocity maneuvers in highly viscous fluid environments. Two examples are the flow disturbances in the cruising of *Metridia longa* and the feeding currents in *Temora longicornis* creating a flow near the surface of the animal body in the direction of animal motion^48^.

Due to the orientation of *E. superba* during the tail flip in this data set, the fluid flow was not visible near the carapace of the animal to confirm or deny the presence of the hypothesized “squeeze” force jet that was first suggested by Daniel and Meyhöfer^37^. Hunyadi et al*.* also did not report evidence of a “squeeze” force^6^.

### Form drag

It is well documented that the caridoid escape mechanism is energetically taxing^7, 37, 49, 50^. Measurements of thrust forces produced for this tail flipping maneuver have been estimated to be 0.42 N in crayfish^6^ and 5–20 N in the spiny lobster *Panulius interruptus*^3^. In an intermediate *Re* flow environment such as that for *E. superba*, there is evidence that the drag force heavily influences the efficiency of the animal’s movement. Kils mentions that Antarctic krill, like many other crustaceans, are able to perform the tail flipping mechanism numerous times in sequence^36^. Kils further notes that although the average swimming speed can be around 50 cm/s over a distance of at least 50 cm, the maximum speed during one tail flip was measured to be 35–100 cm/s^36^. In the current findings, after the tail flip, the animal speed increases to a maximum of 57 cm/s (17.3 BL/s), has a small decrease, and then maintains an average animal speed of 45 cm/s (13.6 BL/s) until the animal travels out of the observation region.

One fascinating result from the hydrodynamic analysis of the flow field is the complex string of vortical structures in the wake after the animal has completed its tail flipping maneuver. Hunyadi et al*.* also reported the presence of a prominent vortical structure behind the tips of the crayfish tail during the abdominal closure and tail flip^6^. This chain of vortices in the wake is reminiscent in structure to the vortex street formed by undulating fish^51–53^. In contrast to these fish wake studies in which the jet flow moves in the direction opposite of the animal motion, the jet flow in the wake of the krill caridoid escape maneuver is in the direction of the animal motion. This flow pattern is similar to a bluff body in a free stream flow field, indicating a suction force being produced due to the rapid movement of the animal.

There is also evidence that the krill is attempting to reduce the drag force to prevent momentum loss. For example, the animal begins closing its tail fan only after the abdominal closure is completed (around t* = 0.75; Fig. 2b). This sequencing maximizes the surface area of the tail during the mechanism presumably to maximize the thrust needed to effectively propel itself backwards. This finding is consistent with Hunyadi et al*.* who performed hydrodynamic studies on the tail flipping mechanism in crayfish^6^. In the animal velocity time record, there is a small dip after the animal reaches its maximum speed before the velocity levels out for the duration of the maneuver (around t* = 0.69; Fig. 2c). However, over the velocity plateau time interval (t* = 0.75–0.9), the tail width of the animal is decreasing. This interval occurs during the abdominal closure period, hence at the beginning of the abdominal closure (t* = 0.62), the tail is still at its maximum width. It is likely that the speed decrease (at t* = 0.69) is due to the drag on the body as the animal begins to accelerate while the tail is still fanned. The animal most likely closes its tail (t* = 0.75–0.9) to reduce drag as the animal begins its glide phase after the abdominal closure, which presumably leads to the leveling of the animal’s speed after the abdominal closure is complete.

### Species comparison

There are a select few studies reporting the kinematics of a caridoid escape response in similar-sized animals. Supplementary Table S1 compares the kinematic parameters of the caridoid escape response between *C. crangon*, *Pandalus danae*, and *E. superba*. The krill data show similar maximum velocity values and distance traveled based on one tail flip as *C. crangon*, but these values are much smaller compared to the *P. danae* kinematics. One interesting note for *P. danae* is that because the glide phase is so long compared to the tail flipping phase of the maneuver, there is usually a reduction in speed of the animal due to form drag^37^. A reduction in speed is also noted for *C. crangon*^47^. However, a similar reduction in velocity is not observed in *E. superba*. Based on dry and wet weight estimates, *E. superba* are less dense than *C. crangon*, most likely due to the characteristics of their exoskeleton^36^. These krill experiments were completed in December, which is summertime the southern hemisphere, and Antarctic krill have been shown to adapt their energy consumption to survive the harsh winters. In the laboratory, Antarctic krill have been shown to survive over 211 days without food and have been reported to fluctuate in weight and lipid contents by up to 4 percent throughout year depending on the season^12^. This large range in mass and body composition may affect the efficiency of the maneuver.

### Wake signature and vortex structure formation

**Figure 9:**
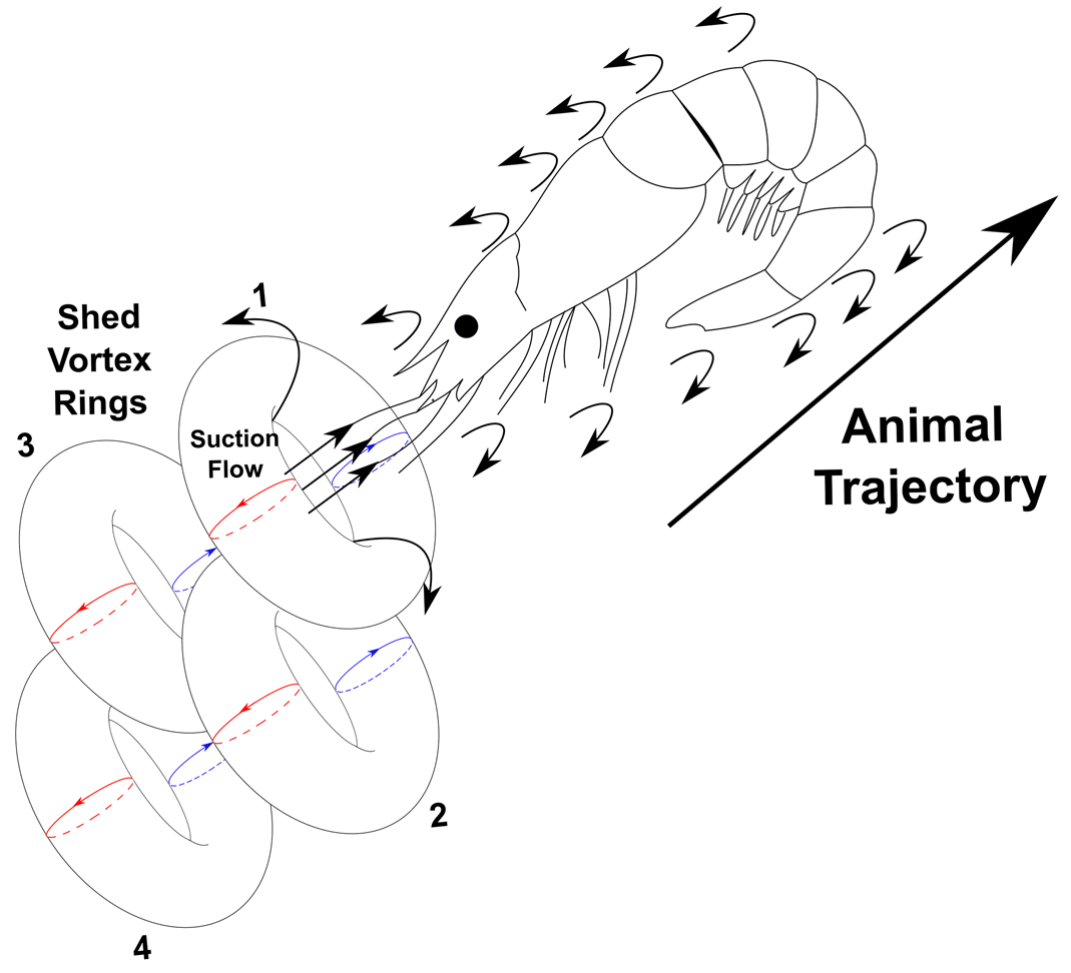
A sketch of the flow field produced by *E. superba* after it has performed its abdominal closure and increased its velocity. As the animal propels itself backwards it generates fluid shear at the surface of the body. The sheared fluid detaches and rolls up from the tip of the antennae to form a trail of vortex rings in its wake.

In the wake of the animal antennae after it completes its abdominal closure and accelerates, *E. superba* generated an extensive and well-defined chain of vortex rings. A sketch representing the different components of the flow characteristics produced by *E. superba* after completing the abdominal closure and accelerating is shown in Fig. 9, based on the reported results. As the animal propels itself backwards, it encounters fluid shearing due to the no-slip condition. At the tip of the antennae, the fluid shear layer converges with the suction flow that is produced from the movement of the animal through the fluid to form a trail of vortex rings in its wake. Jet formation has been reported in several other planktonic crustaceans including the pteropod, *Limacina helicina antarctica*, which forms vortex rings in the wake during both the power and recovery strokes of their parapodia or “wings”^38^. Vortex rings have also been identified in the flow field of the water flea, *Daphnia magna*, which forms viscous vortex rings below its antennae during the power stroke^41^.

The continuous chain of vortex rings found in the caridoid escape maneuver in this study is the first directly measured 3D vortex ring and the first report of a continuous chain of vortex rings shed from the surface of a planktonic crustacean. This finding supports the hypothesis that vortical formations play a significant role in drag-based propulsion at this length scale where the viscous forces of the fluid are significant enough to affect the flow behavior and thus the animal behavior^54, 55^.

## Supplementary Information

Supplementary Information 1.

Supplementary Video 1.

## Data Availability

Data will be provided upon reasonable request addressed to D.R.W (email: dwebster@ce.gatech.edu).
