# Supplementary material for: Hydrodynamics of the fast-start caridoid escape response in Antarctic krill, Euphausia superba
Source: Sci Rep. 2023 Apr 2;13:5376. doi: 10.1038/s41598-023-31676-8 (PMC10068603; doi:10.1038/s41598-023-31676-8)
Supplement: Supplementary file 1 — Supplementary Information 1. [file 41598_2023_31676_MOESM1_ESM.docx]

**Supplementary Table S1.** Species comparison of kinematics data during a caridoid escape response.

|  | Current data | Arnott *et al.*^7^ | Daniel & Meyhöfer^37^ |
| --- | --- | --- | --- |
| Species | *E. superba* | *C. crangon* | *P. danae* |
| Body length (cm) | 3.3 | 1.1 – 6.9 | 7.0 |
| Maximum velocity (cm/s) | 57 | 59 – 231 | 300 |
| Maximum velocity (BL/s) | 17.3 | 25 – 60 | 43 |
| Displacement per tail flip (BL) | 1.3 | 1.4 – 1.8 | 2.1 |
| Reynolds number | 1800 – 11,000 | 6000 – 100,000 | 110,000 – 160,000 |


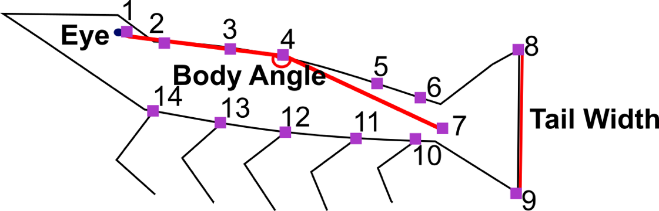


**Supplementary Figure S1.** Diagram of the tracking points defined on the E. superba specimen for kinematic analysis (from the side view). Point 1 represents the eye of the animal, points 2-6 represent the segmented regions along the dorsal side of the abdomen, points 7-9 correspond to the base, right tip, and left tip of the tail, respectively, and points 10-14 represent the locations where each of the five pleopod protopodites meets the abdomen. Body angle is defined as the angle between 2 vectors (shown as red lines) connecting the eye (point 1), the middle of the dorsal side of the abdomen (point 4), and the base of the tail (point 7). Tail width is defined as the distance between the right (point 8) and left (point 9) tips of the tail.


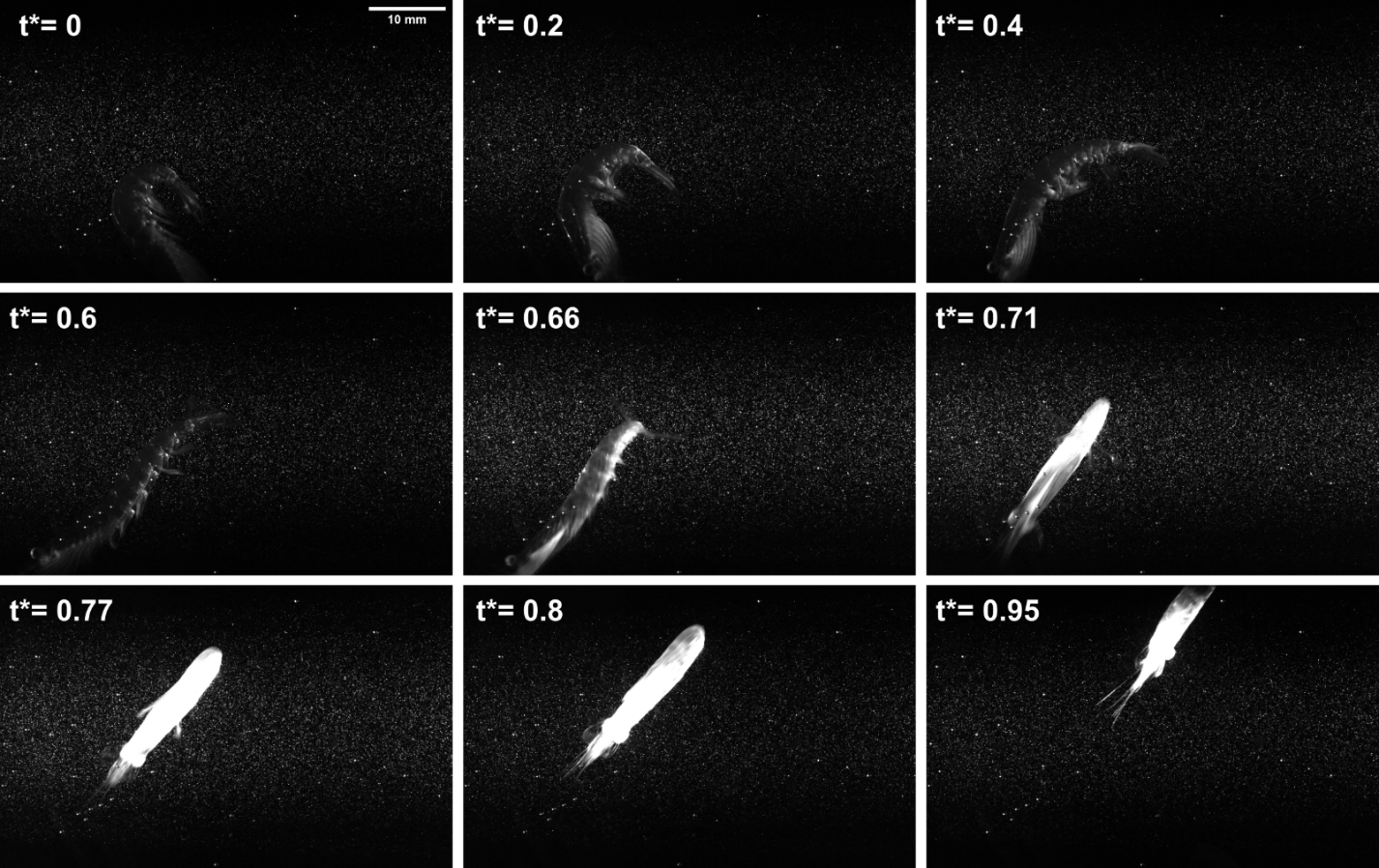


**Supplementary Figure S2.** Time sequence of images of *E. superba* (from a single camera) performing the caridoid escape response where *t** is the time point non-dimensionalized by the total stroke period, which is 322 ms. A 10 mm scale bar is shown in the *t** = 0 frame. The animal starts in a closed body position, fully extends the ventral cavity (*t** = 0 - 0.62), and then performs a rapid abdominal flexion, body rotation, and tail flip (*t** = 0.62 - 0.8) that leads to rapid acceleration of the animal to the upper right section of the image (*t** = 0.66 - 1.0).
